# A physiologically-based flow network model for hepatic drug elimination III: 2D/3D DLA lobule models

**DOI:** 10.1186/s12976-016-0034-5

**Published:** 2016-03-03

**Authors:** Vahid Rezania, Dennis Coombe, Jack A. Tuszynski

**Affiliations:** Department of Physical Sciences, MacEwan University, Edmonton, AB T5J 4S2 Canada; Computer Modelling Group Ltd, Calgary, AB T2L 2A6 Canada; Department of Physics and Experimental Oncology, University of Alberta, Edmonton, AB T6G 2J1 Canada

## Abstract

**Background:**

One of the major issues in current pharmaceutical development is potential hepatotoxicity and drug-induced liver damage. This is due to the unique metabolic processes performed in the liver to prevent accumulation of a wide range of chemicals in the blood. Recently, we developed a physiologically-based lattice model to address the transport and metabolism of drugs in the liver lobule (liver functional unit).

**Method:**

In this paper, we extend our idealized model to consider structural and spatial variability in two and three dimensions. We introduce a hexagonal-based model with one input (portal vein) and six outputs (hepatic veins) to represent a typical liver lobule. To capture even more realistic structures, we implement a novel sequential diffusion-limited aggregation (DLA) method to construct a morphological sinusoid network in the lobule. A 3D model constructed with stacks of multiple 2D sinusoid realizations is explored to study the effects of 3D structural variations. The role of liver zonation on drug metabolism in the lobule is also addressed, based on flow-based predicted steady-state O_2_ profiles used as a zonation indicator.

**Results:**

With this model, we analyze predicted drug concentration levels observed exiting the lobule with their detailed distribution inside the lobule, and compare with our earlier idealized models. In 2D, due to randomness of the sinusoidal structure, individual hepatic veins respond differently (i.e. at different times) to injected drug. In 3D, however, the variation of response to the injected drug is observed to be less extreme. Also, the production curves show more diffusive behavior in 3D than in 2D.

**Conclusion:**

Although, the individual producing ports respond differently, the average lobule production summed over all hepatic veins is more diffuse. Thus the net effect of all these variations makes the overall response smoother. We also show that, in 3D, the effect of zonation on drug production characteristics appears quite small. Our new biophysical structural analysis of a physiologically-based 3D lobule can therefore form the basis for a quantitative assessment of liver function and performance both in health and disease

**Electronic supplementary material:**

The online version of this article (doi:10.1186/s12976-016-0034-5) contains supplementary material, which is available to authorized users.

## Background

The liver is a complex organ that removes chemicals, including drugs, from the blood through metabolic processes. When such liver detoxification processes are incomplete or overwhelmed, liver damage can result leading to liver failure. Drug access to hepatocytes is governed by transport processes in the well-vascularized liver tissue, and so structural variability can impact such transport. Therefore, a quantitative understanding of drug distribution and metabolism in the liver is essential for the ability to predict both liver performance and damage. We envisage a future generation of algorithms for anatomically- and physiologically-based liver models that can be then used for the prediction of optimal doses and scheduling of various drugs prior to clinical administration. This could be especially useful for patients with liver diseases such as hepatocellular carcinoma or cirrhosis.

Several investigators have recently explored computational fluid models of liver lobule function. Ierapetritou et al. [1] give a comprehensive general overview of the modelling approaches and issues that arose up to 2009. More particularly, Rani et al. [2] developed a detailed computational dynamics model of a small portion of a liver lobule, focusing on the non-Newtonian characteristics of blood. They considered the flow along one sinusoid with exit fenestrations fed by a portal vein and hepatic artery segments and exiting via a hepatic vein segment. The contributions of portal vein (PV) versus hepatic artery (HA) flows to the overall pressure drop and velocity profiles were detailed, including regions of Eddie flows and high strain rates near the exit fenestrations. Steady state flows were achieved after 5 × 10^−5^ s.

Yan et al. [3] developed a physiologically-based, multi-agent model of liver lobule performance. They used a Monte Carlo selection method to determine properties of the sinusoidal graph structure, convective-dispersive flow and metabolic interactions. This allowed inclusion of lobule zonation effects plus the metabolic influences of chemical molecular weight, octanol partition coefficients and protein binding to predicted drug-liver lobule interactions. They matched lobule outflow characteristics of 4 cationic drugs (atenolol, antipyrine, labetalol, and diltiazem), using sucrose as a base flow chemical. Wambaugh and Shah [4] compared the role of various lobule (sinusoidal) morphologies on drug propagation again using an agent-based simulation approach. While demonstrating that their model can reproduce traditional coarse averaging results (well-mixed and parallel tubes) under some flow regimes, they emphasized the utility of their random statistical approach for rapidly metabolized chemicals.

Hoehme et al. [5] employed a combined experimental and computational study of lobule structural restoration after CCl_4_ damage over several days as a protocol for liver regeneration. They used agent-based representations of both hepatocytes and sinusoids in their computational model and demonstrated that the regeneration process is characterized by a hepatocyte-sinusoid alignment process to correctly restore liver microarchitecture. Schliess et al. [6] extended this approach to consider ammonia detoxification during liver damage and regeneration. Here they first proposed a simple metabolic model considering ammonia, urea, and glutamine components utilizing three mass balance ordinary differential equations (ODEs) for chemical reactions and two compartments (periportal and perivenal) such that urea generation is maximized in the periportal region while glutamine regeneration occurs perivenally. The resulting concentrations were interpolated on their spatial-temporal grid as the sizes of the damaged zones change over the regeneration process. They also considered a multi-lobule pattern to reduce boundary effects. Drasdo et al. [7] summarized both modeling approaches in a review article.

**Fig. 1:**
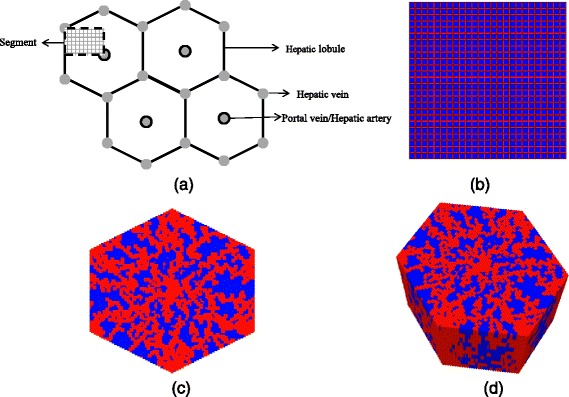
Various flow network structures. **a** Schematic diagram of a cross section of hepatic parenchyma consisting hexagonal lobules, portal and hepatic veins. The lobule contains sinusoids and liver cells (hepatocytes). The segment represents a typical area studied in our previous papers [8, 9]. **b** Homogeneous lattice (segmented area) with high porosity bands (in red) representing sinusoids and lower porosity regions (in blue) representing tissue containing hepatocytes, as in our previous papers [8, 9]. **c** 2D lobule lattice with sinusoids generated via a diffusion limited aggregation algorithm, this paper. **d** 3D lobule lattice, this paper

In previous papers (papers I [8] and II [9]), we proposed a physiologically-based lattice model to study the transport and metabolism of drugs in the liver lobule. In these studies, we constructed a simple regular square lattice model that represents a portion of a liver lobule, see Fig. 1a and b, to explore the dynamics of competing convective, diffusive, and reactive processes acting on an injected chemotherapeutic drug paclitaxel. Structural and spatial variations and liver lobule zonation were also considered and their impacts on the hepatic drug metabolism were discussed. Such simulations had the useful consequence of interpreting drug concentration levels found exiting the lobule in terms of their detailed spatial distribution within the lobule, caused by competing processes. This analysis forms the basis, and a point of contrast, to the drug distributions obtained when some of these basic assumptions on lobule structure are relaxed.

In this paper, we extend our biophysical structural analysis to a full lobule situation, a hexagonal based model with one central blood in-flow (portal vein) and six corner located out-flows (hepatic veins), see Fig. 1. We also introduce the sinusoid network using a sequential diffusion-limited aggregation (DLA) algorithm (as presented and discussed below, Fig. 3) to gain insight on a more realistic resulting morphological sinusoid structure, see Fig. 1c. In a nutshell, sequential means a series of DLA generated pattern steps (5 steps) to generate the desired sinusoid structure variations in 2D. Thereafter we repeat this algorithm for various layers to generate a representative 3D lobule pattern. As a result, individual sinusoid layers, although generated by the same DLA algorithm, are not identical but exhibit some variability as observed in real lobules. A sensitivity analysis is conducted by observing drug concentration levels exiting the lobule with their predicted detail distribution inside the lobule.

### The liver lobule functional unit

It is commonly accepted that a single lobule serves as the functional unit of the liver, the smallest structural unit of the liver with ability to perform all hepatic functionalities [10]. The classic lobule is a hexagonal cylinder, centered around a hepatic venule and with portal tracts situated at the corners. The portal lobule has a similar shape but is centered about a portal tract with the hepatic venules at the periphery [11]. We shall invoke this second point of view as the basis for our model development.

Even among the approximately 1.5 million lobules (assuming a liver size of 1500 cm^3^ and a lobule size of 1 mm^3^) that make up the human liver, structural variability of lobule units is the rule. Teutsch and colleagues [12] illustrate this specific microarchitecture variability and diseased states can be expected to add additional variability. Here we will attempt to quantify the consequences of such variability via our computational model.

## Methods

### 2D hexagonal lobule construction

The flow equations describing reactive-convective-diffusive flow in the liver lobule remain unchanged from our first two papers. Figure 1a and b illustrate the network structure of our base case model discussed in papers I [8] and II [9] (which uses a regular sinusoid pattern). Figure 1c represents our model that we study here, which consists of a 2D square lattice clipped by hexagonal boundary with DLA constructed sinusoid network. Figure 1d demonstrates a 3D extension of the lobule model that will be discussed later.

**Fig. 2:**
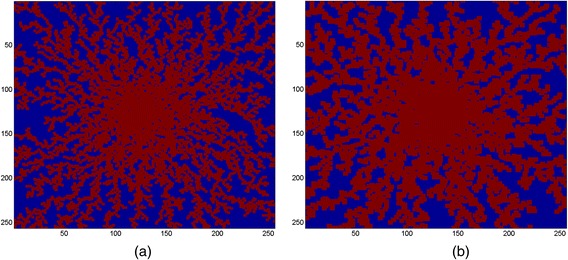
Diffusion aggregated runs. **a** *N*_par_ = 14,000 and *dp* = 1 pixel, **b** *N*_par_ = 7000 and *dp* =2 pixel. Here blue dots have value of zero representing no particle is on that site (to be used as hepatocytes) and red dots have value of 1 representing a particle is on the site (to be used as sinusoids)

In this paper, MATLAB [13] has been used to code and generate sinusoid network using a novel sequential DLA algorithm. We use a standard DLA algorithm that randomly clusters a specified number of particles (here *N*_par_) with particle size (*dp* × *dp* in a pixel^2^). Figure 2a and b show result of DLA runs for *N*_par_ = 14,000 and *dp* = 1 pixel and for *N*_par_ = 7000 and *dp* = 2 pixel, respectively.

**Fig. 3:**
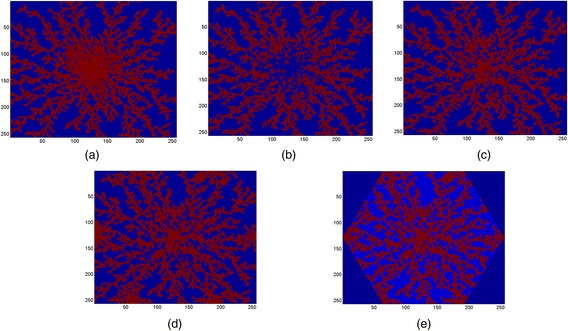
Various steps to create a DLA hexagonal lobule. **a** Main DLA pattern with *N*_par_ = 9500 particles and size *dp* = 1 on a domain *M*_dim_ = 256, **b** de-crowding the central region using a DLA pattern, **c** filling the central region again with a DLA pattern, **d** creating corner wells using a DLA pattern, **e** eliminating exterior points of the hexagon
ii) As shown in Fig. 3a, the central region is very crowded. We overwrite the central region (here we chose half-the original field) using the DLA algorithm and with smaller number of zero-valued particles (blue dots). Here we use *N*_cen1_par_ = 1200, *M*_dim_ = 128 and *dp* = 1. See Fig. 3b.
iii) To fill the central region with sinusoids again (red dots), the DLA algorithm will be called for the third time with fewer particles, i.e. *N*_cen2_par_ = 800. Here *M*_dim_ = 128 and *dp* = 2. See Fig. 3c.
iv) In this step, we determine the six corners of the hexagon to create the out-flowing wells at these locations. Again the DLA algorithm will be used to create a DLA pattern around each corner. See Fig. 3d. Here we use the domain size of 1/3 of the original domain, i.e. *M*_dim_/3, and fewer particles, *N*_cor_par_ = 500. Here *dp* = 1.
v) At the final stage, any point outside the bounding hexagon is removed. Figure 3e shows the final result if the DLA hexagonal lobule. As shown, hepatocytes are blue islands that are encompassed by red sinusoids.

In order to create a hexagonal lobule comparable to a realistic liver lobule, five steps are taken:

i) A zero *M*_dim_ × *M*_dim_ matrix (field) has been created and the DLA algorithm has been called to create a DLA pattern with specified *N*_par_ and *dp*. A particle, valued 1, has been located at a random position in the field by the DLA algorithm and then performed a random walk (with a desired step size, here denoted by *dp*) toward the center of the field. The newer particle will do the same until it hits another (older) particle, sticks to it and stops. This will continue for all *N*_par_ = 9500 particles. See Fig. 3a. Here *M*_dim_ = 256 and *dp* = 1.

This sinusoid generation algorithm is similar in spirit but not in details to that employed by Wambaugh and Shah [14]. They have used the term “sinusoid morphology” to characterize these investigations, which we will also employ. In recent work, Hoehme et al. [15] captured realistic lobule sinusoid patterns from image analysis of confocal microscopy of liver tissue. To check whether our simulated sinusoid pattern is comparable with their result, we perform a population density analysis for several 2D simulated and real lobules (Wisk S, Rezania V: A comparison between real and DLA simulated liver lobules using a population density analysis, Submitted). We calculated the ratio of the area covered by sinusoids to the total area of the lobule for both simulated and real 2D lobule images to optimize values of *N*_par_ and *dp* for corresponding *M*_dim_. Based on these calculations, we find *N*_par_ ≈ 37 *M*_dim_ (~9500) and *dp* = 1 will produce comparable results (~45 % sinusoid area/total 2D area). A comparison of our Fig. 3d with their results demonstrates that our DLA algorithm with the chosen parameters generates equally physiologically reasonable sinusoid morphology patterns (in particular, see their Fig. 1d or further details in their SI Appendix, their Fig. 3d).

### Extension to a 3D lobule structure

In order to generate an even more realistic survey, we next study a 3-dimensional lobule. Again our morphological sinusoid generation algorithm is similar in spirit to that employed by Wambaugh and Shah [14] in their 3D models.

**Fig. 4:**
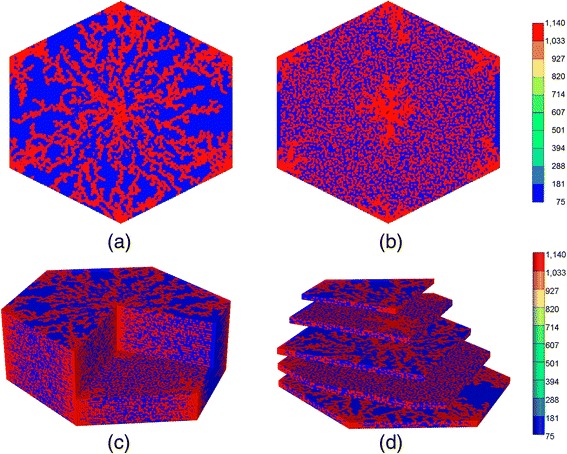
Three-Dimensional DLA hexagonal lobule for *M*_z-dim_ = 97 and *M*_skip_ = 2. **a** A 2D DLA (sinusoid) layer, **b** a skipped (hepatocyte) layer, **c** 3D cutaway view **d** 3D clipped view
iii) Then, two DLA layers vertically will be connected at several random locations to approximate the shape hepatocyte cells, see Fig. 4b.
iv) The center and the six corners of the hexagon in all layers will be connected to represent arterial and portal veins.

The 3D DLA structure is essentially constructed by stacking several 2D DLA structures as follows:

i) A vertical dimension *M*_z-dim_ will be specified.
ii) Starting from the very bottom layer that has a 2D DLA structure, we skip *M*_skip_ layers to introduce the next layer with a 2D DLA structure (a DLA layer), see Fig. 4a. This procedure will be repeated to reach to the very top layer which again is a DLA layer. Here, each DLA layer represents the sinusoidal network. Two of these layers encompass hepatocyte cells (the skipped layers). The value of *M*_skip_ is chosen based on the average thickness size of a hepatocyte cell.

Since the skipped layers represent the hepatocyte tissue, the value of *M*_skip_ will be determined based on the typical volume of a lobule and hepatocytes. The typical volume of a human liver lobule is somewhat less than 1 mm^3^ [12]. Furthermore, a hepatocyte has a diameter of 12 – 24 μm and thickness of 25 μm in average and mean sinusoid thickness is about 5 – 7 μm. Here, we chose a sinusoidal cell as a cube with dimension of 6 μm and a hepatocyte cell as rectangular cube with dimensions 6 μm and 12 μm, with thickness of 6 μm in the DLA layers and 12 μm in the skipped layers, respectively. The area of a hexagon with diameter of *d* is given by $S=3\sqrt{3}{d}^2/8$.

In our case, *d* = 256 × 6 μm = 1536 μm that leads to *S* = 1.53 × 10^6^ μm^2^. The 3D lobule is then composed by stacking up 33 sinusoidal layers and 64 tissue layers (*M*_z-dim_ = 97) that gives an approximate model volume of 1.5 mm^3^. Here, every two tissue layers are sandwiched by two sinusoidal layers (*M*_skip_ = 2). This choice of tissue/sinusoid grid thickness represents a compromise between simulation runtime speed and numerical discretization error.

Figure 4 shows the 3D DLA hexagonal lobule for *M*_z-dim_ = 97 and *M*_skip_ = 2. Figure 4a demonstrates a 2D DLA layer representing a sinusoidal layer. Figure 4b shows a hepatocyte layer. The red points connect all hepatocyte layers between two DLA layers. These points approximately determine the boundary of the hepatocyte cells. To construct the third dimension, a DLA layer is created for the bottom, then two hepatocyte layers (*M*_skip_ = 2), and then a new DLA layer will be produced. The procedure repeats over the whole *z*-dimension. Figures 4c and d show two different 3D views.

### Flow calculation methods

Convection-diffusion-reaction flow calculations are performed on the generated models utilizing the STARS advanced process simulator [16] as described in our earlier papers. Here we have chosen paclitaxel (PAC) [17] as our example drug, as it is one of the most widely used chemotherapy agents, especially active against many human solid tumors [18] (breast cancer, ovarian cancer, lung cancer, etc). Table 1 summarizes our base case flow and metabolic parameters for PAC (from Vaclavikova et al. [19]) used in these calculations for a representative human lobule. Cytochrome P450 CYP2C8 or CYP3A4 [20] are the enzymes active in PAC metabolism. Due to the non-regular areal sinusoidal structures generated here, however, it was found necessary to utilize a higher order discretization method (“areal 9-point”) to produce the expected smooth profiles on these grids. A discussion of this (quite standard) discretization method can be found in the STARS manual.

**Table 1:** Base case flow and metabolism parameters

Parameter	Characteristic (SI) Unit	STARS Unit
Sinusoid Porosity *ϕ*_sin_	0.7854	0.7854
Sinusoid Permeability *K*_sin_	1.125 μm^2^	1.140 Darcy^a^
Sinusoid Effective Diffusion *D*_sin_	4.2 × 10^−10^ m^2^/s	2.5 × 10^−4^ cm^2^/min
Tissue Porosity *ϕ*_tis_	0.2382	0.2382
Tissue Permeability *K*_tis_	7.35 × 10^−2^ μm^2^	7.45 × 10^−2^ Darcy
Tissue Effective Diffusion *D*_tis_	4.2 × 10^−11^ m^2^/s	2.5 × 10^−5^ cm^2^/min
Maximum Rate *v*_max_^b^	0.06 μM/min	1.08 × 10^−9^ mole fraction/min
Half Saturation Constant *K*_m_^b^	10.0 μM	1.8 × 10^−7^ mole fraction
Linear Rate *v*_max_/*K*_m_^b^	6.0 × 10^−3^ min^−1^	6.0 × 10^−3^ min^−1^
Blood Viscosity *μ*	3.5 × 10^−3^ Pa-sec	3.5 cpoise

^a^ 1 Darcy = 0.9869 μm^2^ in engineering permeability units

^b^ PAC kinetic elimination Michaelis-Menten parameters converted from Vaclavikova et al. [17], their Table 4

## Results and Discussion

### 2D drug distribution – comparison to our earlier studies

**Fig. 5:**
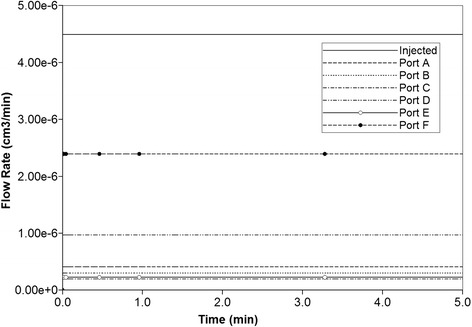
Injected flow (PAC). Steady state flow across the 2D lobule

Similar to previous studies, flow is induced in the lobule lattice by applying a pressure difference across the central inlet and six corner outlet points. With the chosen lobule flow parameters for porosity, permeability, and blood viscosity, the steady flow rate is 4.491× 10^−6^ cm^3^/min with (0.4077, 0.3011, 0.1950, 0.9682, 0.2251, 2.394) × 10^−6^ cm^3^/min for ports A to F, respectively, as illustrated in Fig. 5. (For steady flows, the inflow rate equals the total outflow rate.) It is clear that ports D and F have highest outflow among the others. This is due to the random nature of the DLA simulation for this realization.

**Fig. 6:**
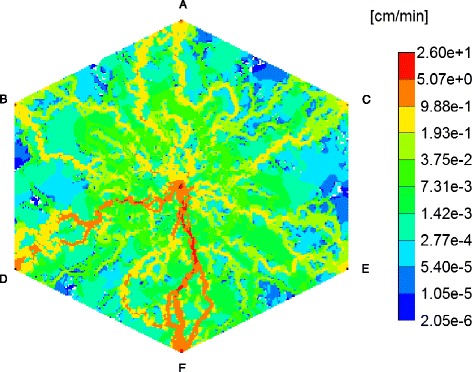
Steady state velocity profile across the 2D lobule. Color bar is in cm/min

Figure 6 demonstrates the steady state velocity profile throughout the lobule lattice, illustrating both the diverging/converging nature of the flow near the inlet and outlet ports (i.e. injector and producers, respectively), as well as the orders of magnitude difference of the flows in the sinusoids and tissues, respectively. (This plot uses a logarithmic colour scale axis). Ports D and F show highest velocities among the ports.

**Fig. 7:**
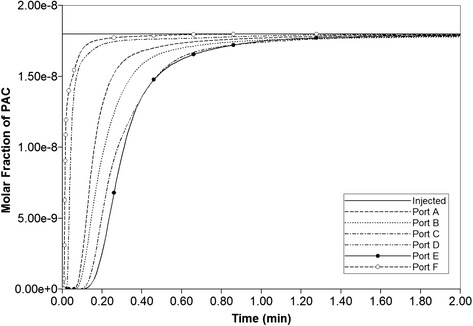
Non-reactive PAC drug propagation across the lobule without diffusion effects

Blood with a relative composition of 1 microgram PAC (1.8 × 10^−8^ mole fraction) is infused into the lobule through the central inlet. Assuming nonreactive hepatocytes, the time required to traverse the lattice is approximately 1 min or less as demonstrated in Fig. 7. The fastest drug propagation is through ports D and F for this realization. Similar to our Paper I [8], this production profile is convective flow dominated as the addition of diffusion minimally alters the production profile (figure not shown).

As indicated in our Paper II [9] concerning variable lattices models with multiple realizations, flow induced by the application of a fixed pressure drop across a lobule model can be expected to vary significantly for each model realization. This will in turn impact on the propagation and production characteristics of both non-reactive and metabolized drugs. Thus multiple realizations of the morphological sinusoidal network generated by our DLA algorithm here induce a similar variability in flow and drug production behavior. Table 2 summarizes the flow variability for 16 such realizations. In what follows, we will focus on the analysis of drug behavior for one such realization.

**Table 2:** Flow rate variability of multiple 2D DLA sinusoid morphologies

Layer no.	SS flow rate (×10^−5^ cm^3^/min)
Lyr1	1.824
Lyr4	1.366
Lyr7	1.829
Lyr10	0.776
Lyr13	1.781
Lyr16	1.716
Lyr19	1.399
Lyr22	0.889
Lyr25	1.555
Lyr28	1.225
Lyr31	1.684
Lyr34	1.416
Lyr37	1.251
Lyr40	1.044
Lyr43	1.421
Lyr46	1.983
Average	1.447
STDEV	0.3511

**Fig. 8:**
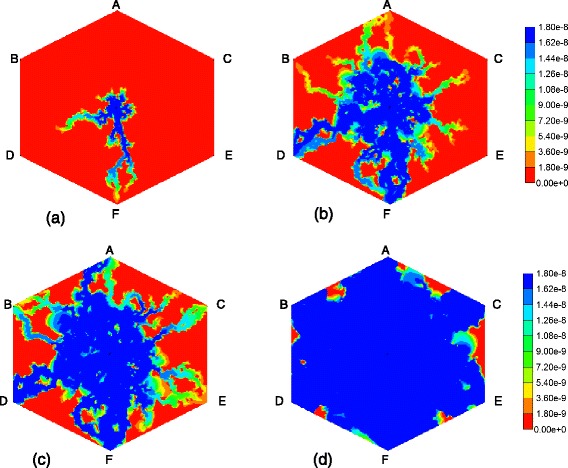
Non-reactive PAC profiles across the lobule without diffusion. **a** PAC at 0.01 min, **b** PAC at 0.1 min, **c** PAC at 0.2 min, **d** PAC at 5 min. Color bar is in mole fraction

**Fig. 9:**
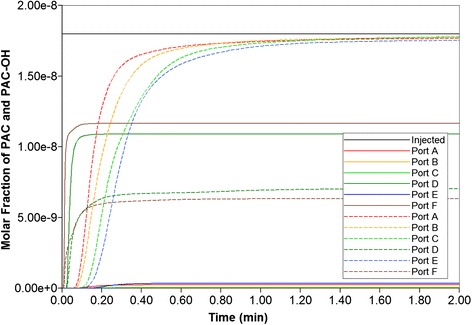
Reactive (6 × 10^−6^ min^−1^) PAC (solid lines) and PAC-OH (dashed lines) drug propagation across the lobule, without diffusion effects and base case metabolism

The spatial progression of the PAC concentration on the lobule lattice without and with diffusion is demonstrated in Figs. 8 and 9, respectively. The diffusion constants for PAC and PAC-OH are estimated as 2.5 × 10^−4^ cm^2^/min in sinusoid and 2.5 × 10^−5^ cm^2^/min in tissue, respectively. In the absence of diffusion, only a small “pressure-difference-driven (convective)” transfer from sinusoids to tissue through the space of Disse is occurring. See our Paper I [8] for details.

Figures 8 and 9 show the increasing levels of injected drug from 0.01 min to 5 min (0.5 min in case with diffusion). As shown, by 0.01 min, PAC almost reaches the outgoing port F of the lobule.

In case with no diffusion, it takes almost 5 min to fill the lobule (Fig. 8), while PAC completely covers the lattice after 0.5 min when diffusion is on as demonstrated in Additional file 1: Figure S1.

### Reactive flows in 2D

Now we consider the effects of PAC drug metabolism by hepatocytes. Here the base case reaction parameters of Table 3 of Paper I [8] are employed, and the same injected PAC concentration (1.8 × 10^−8^ mole fraction) is considered. With the employed reaction half saturation constant value of 1.8 × 10^−7^ mole fraction, this injection level implies the Michaelis-Menten model reduces to an almost linear reaction equation.

Figure 9 illustrates injected drug and produced drug and metabolite production for this case. The reaction rate is 6 × 10^−3^ min^−1^. Again it is emphasized that both PAC and PAC-OH have assumed equal diffusive flow contributions, as these are components of very similar size. Essentially at this reaction rate, all injected PAC is converted to metabolite by the lobule hepatocytes. The production profile of PAC-OH here is similar to the production profile of PAC in the non-reaction case, as shown in Fig. 7, for most of the ports. That is, the reactive conversion of PAC to PAC-OH occurs reasonably quickly. However, for the two ports with higher flows (ports D and F), less conversion of PAC to PAC-OH is seen to occur.

**Fig. 10:**
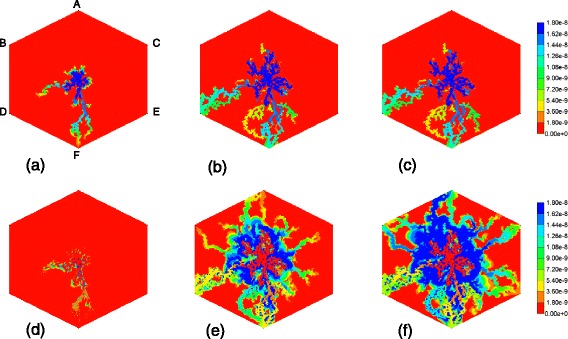
Reactive (6 × 10^−6^ min^−1^) PAC and PAC-OH profiles across the lobule without diffusion effects and base case metabolism. **a** PAC at 0.01 min, **b** PAC at 0.10 min, **c** PAC at 0.20 min, **d** PAC-OH at 0.01 min, **e** PAC-OH at 0.10 min, **f** PAC-OH at 0.20 min. Color bar is in mole fraction

Figure 10 shows the PAC and PAC-OH profiles across the lobule lattice at 0.01 min, 0.10 min, and 0.20 min, respectively. The PAC concentrations in the sinusoids and the PAC-OH concentrations in the tissue are equivalent to the PAC concentrations in both sinusoids and tissue for the non-reacted case (Fig. 8). Note that after 0.1 min there is no change in PAC spatial distribution as it converts to PAC-OH before propagating further. Figure 10 also shows clearly there is an inlet distance over which the reaction conversion time is not fast enough to convert the injected PAC.

**Fig. 11:**
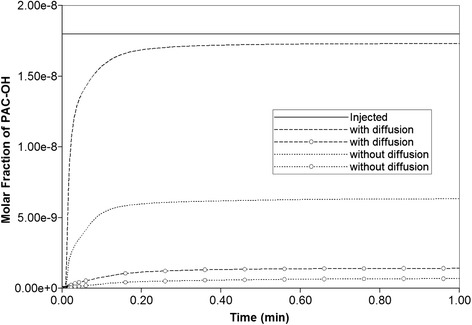
Reactive PAC-OH drug propagation across the lobule, with and without diffusion effects. For two reaction kinetics 6 × 10^−6^ min^−1^ (curves with circle) and 6 × 10^−3^ min^−1^

Diffusion, however, has greater impacts on the reactive cases. Figure 11 compares the overall PAC to PAC-OH conversion for two reactive cases with reaction rate 6 × 10^−6^ min^−1^ and 6 × 10^−3^ min^−1^, with and without diffusion. (The latter rate is our base case which has the higher conversion rate.) As shown, at each rate a greater concentration of PAC-OH is generated when diffusion is on, as this provides an additional mechanism to bring PAC molecules to the reactive hepatocyte sites.

### 3D drug distribution – extension of behaviors

As stated above, the 3-dimensional lobule is constructed by stacking up 33 sinusoidal layers and 64 hepatocyte layers (*M*_z-dim_ = 97) with the approximate volume of 1.5 mm^3^. The sinusoidal layers have similar structures (randomly generated though) as the 2D DLA lobule structure discussed in previous section. The hepatocyte layers are generated by randomly selected points to mimic hepatocyte cell distribution in a lobule. All layers are connected through the central region (the portal vein) as well as six corners of the hexagon (hepatic veins). In this section we are interested in studying the effect of the third dimension as the blood will be injected from the central vein in all layers simultaneously.

**Fig. 12:**
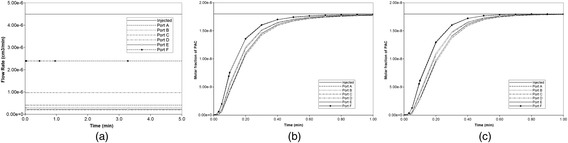
Non-reactive PAC drug propagation across the lobule in 3D. **a** steady state flows across the 3D lobule (**b**) PAC propagation without and (**c**) PAC propagation with diffusion effects

In 3D, the steady flow rate across the full lobule is 3.00× 10^−3^ cm^3^/min as shown in Fig. 12a, as all volumes have been upscaled from our 2D slice models. As seen in Fig. 12b and c, in this realization of the 3D lobule, ports A and F have highest outflow (they are essentially superimposed), then ports B and D (they are essentially superimposed), with C and E having the lowest outflow rates. This is again due to the random nature of the DLA simulation for this realization. However the port-to-port variation in flows is significantly less extreme in 3D, as the third dimension provides additional connecting flow paths to smooth the overall flow pattern.

**Fig. 13:**
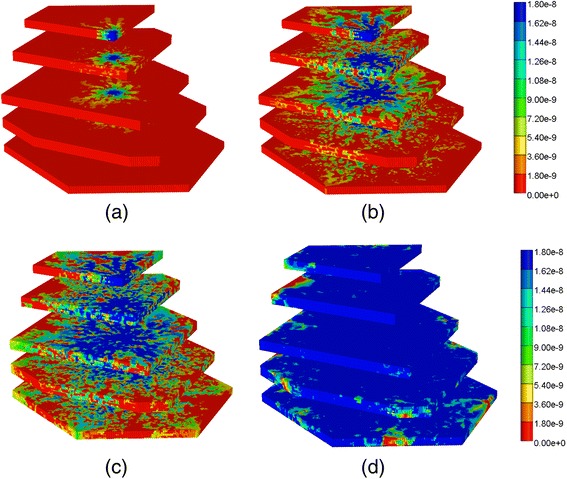
Non-reactive PAC profiles across the lobule without diffusion in 3D – clipped view. **a** PAC at 0.01 min, **b** PAC at 0.05 min, **c** PAC at 0.1 min, **d** PAC at 1 min. Color bar is in mole fraction

Figure 13 gives a spatial view (clipped–view) of the increasing levels of the injected PAC drug from 0.01 min to 1 min across the lobule without diffusion and no reaction. The drug first clearly follows the high flow paths provided by the morphological sinusoid network, and only slowly reaches into the tissue portions of the lobule. A different spatial view (block–view) is also demonstrated in Additional file 2: Figure S5.

The same two views of non-reactive PAC injection with diffusion added are shown in Additional file 3: Figure S2 and Additional file 4: Figure S3. In those figures the sinusoid-tissue transfer rate is much more rapid and PAC drug propagates in a more uniform fashion as it enters the lobule. Clearly, diffusion has a very significant effect on the drug propagation details although at later times both cases result in a uniform drug coverage of the lobule.

### Reactive flows in 3D

Now we consider the effects of PAC drug metabolism by hepatocytes across the 3D lobule. Similar to the 2D case, the same injected PAC concentration (1.8 × 10^−8^ mole fraction) is considered.

**Fig. 14:**
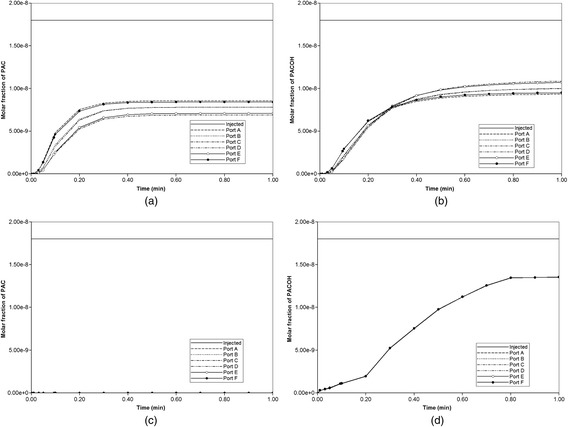
Reactive (6 × 10^−3^ min^−1^) PAC and PAC-OH drug propagation across the lobule in 3D. **a** PAC without diffusion, **b** PAC-OH without diffusion, **c** PAC with diffusion, **d** PAC-OH with diffusion

Figure 14 illustrates injected drug and produced drug and metabolite production for this case without and with diffusion effects. The reaction rate is 6 × 10^−3^ min^−1^. As before, both PAC and PAC-OH have assumed equal diffusive flow contributions.

Figures 14a and b demonstrate the levels of PAC and PAC-OH, respectively, without including diffusion effects. As shown, both PAC and PAC-OH have similar concentrations after 1 min in all outlets (i.e. approximately half of injected PAC is metabolized to PAC-OH). The diffusion, however, alters the latter behavior significantly as depicted in Fig. 14c and d. All the PAC converted to PAC-OH almost immediately. The variability of the produced PAC-OH concentration per port for this reactive case with diffusion mirrors the variability of the produced PAC per port for the nonreactive case (Fig. 11, with or without diffusion).

**Fig. 15:**
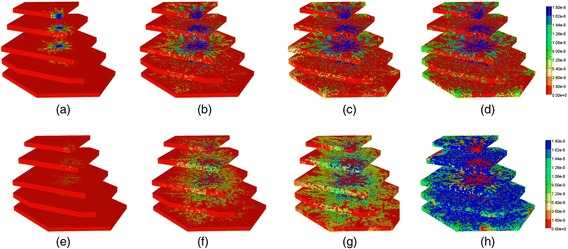
Reactive (6 × 10^−3^ min^−1^) PAC and PAC-OH profiles across the lobule without diffusion effects and base case metabolism in 3D – clipped view. **a** PAC at 0.01 min, **b** PAC at 0.05 min, **c** PAC at 0.1 min, **d** PAC at 1 min, **e** PAC-OH at 0.01 min, **f** PAC-OH at 0.05 min, **g** PAC-OH at 0.10 min, **h** PAC-OH at 1 min. Color bar is in mole fraction

Figure 15 shows the spatial clipped-view of reactive PAC and PAC-OH distributions in the 3D lobule at selected times without diffusion effects and with the base case metabolism rate. As time progresses, more PAC is metabolized to PAC-OH but the metabolic rate is low enough that both PAC and PAC-OH distribute throughout the lobule. This distribution is not uniform throughout the lobule however. For example, there remains an inlet zone with a negligible amount of PAC-OH at all times. The spatial block-view is also demonstrated in Additional file 5: Figure S6.

As shown in Additional file 6: Figure S4, with diffusion but still utilizing a base case metabolic rate, the in-situ conversion of PAC to PAC-OH is much more rapid and uniform, and results in an almost total drug conversion to PAC-OH throughout the 3D lobule. There remains only a small inlet region of unconverted PAC.

As in the 2D cases presented earlier, diffusion has greater impact on drug distribution for the reactive cases. Similar behaviors can be observed for other reaction rates (e.g. reaction rate 6 × 10^−1^ min^−1^) with and without diffusion (not shown).

### 3D drug distribution with zonation

Zonation is a well-known feature of many metabolic processes in the liver lobule [15], some processes are up-regulated in the periportal region, while others are up-regulated in the perivenal region. Here we consider zonation of drug metabolizing enzymes (in particular cytochrome P450 – CYP2C8), such that higher CYP levels are found near the perivenal region.

Zonation has been attributed primarily to a non-uniform distribution of O_2_ across the lobule [16]. Here we will utilize this experimental observation to predict relative CYP levels based on a calculated O_2_ distribution. Appendix A presents details of the O_2_ convective-diffusive-reactive flow problem employed to generate the zoned-CYP distribution.

The resultant initial non-homogeneous distribution of CYP enzymes is imported into the PAC reaction model in a manner analogous to that employed in our earlier idealized model of zonation [9]. Here we consider enhanced perivenal enzyme expression (the “reversed” case of reference [9]) as a reference, where CYP expression is active primarily near the lobule outlet zone. In our present work, two scenarios are envisioned: (a) a CYP distribution generated from “normal” levels of O_2_ injection, and (b) that generated from a “low concentration” O_2_ injection level. The result is shown in Figures A3 and A4, which can be contrasted to Additional file 3: Figure S2b of our earlier idealized model [9]. These distributions are used to modify the PAC metabolic rates non-homogeneously in a manner similar to our earlier paper.

**Fig. 16:**
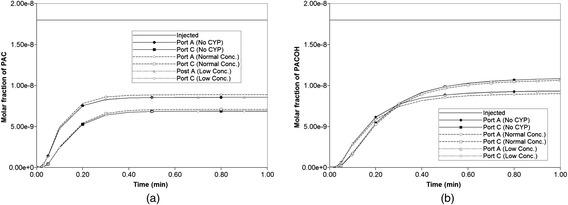
Reactive (6 × 10^−3^ min^−1^) PAC and PAC-OH profiles across the lobule without diffusion effects and normal versus low concentration–zonation metabolism (**a**) PAC production (**b**) PAC-OH production

Figure 16 compares reactive PAC and PAC-OH production profiles with no diffusion contributions to the flow. Note that both PAC and PAC-OH are produced here as in our ideal treatment of zonation found in Paper II [9]. In Fig. 16 only ports A and C are shown as their productions show the largest variation among all six production ports. Zonation (either averaged or extreme) is seen to have a small effect on the production behavior compared to the base case (no zonation) behavior, with the low concentration zonation profiles essentially identical to the no zonation case (see the almost uniform CYP profile generated for this case, Figure A3). Finally, with the same (reversed) zonation patterns, but assuming additional diffusive contributions to the flow, mostly only PACOH is produced, and no differences in production behavior are seen with or without zonation (not shown). This is consistent with our ideal treatment of zonation described in Paper II [9].

Spatial plots of PAC and PACOH distributions at various time points for the no diffusion cases with zonation show only subtle differences from the base case no-zonation profiles of Fig. 16 and thus are not shown explicitly. This is perhaps not surprising for the low O_2_ concentration generation case with a very uniform zoned CYP distribution, but is more puzzling for the normal O_2_ concentration generated case. We believe this is a result of the high PAC to PACOH conversion rate chosen as our example for this paper coupled to the downstream distribution of CYP. This example appears to be quite robust to details of the exact CYP zonation pattern.

**Fig. 17:**
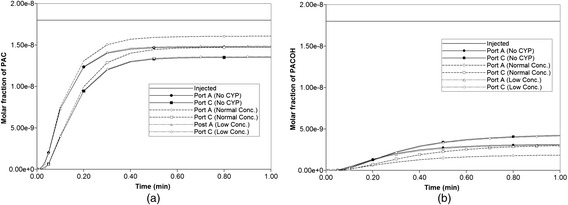
Reactive (6 × 10^−6^ min^−1^) PAC and PAC-OH profiles across the lobule without diffusion effects and normal versus low concentration–zonation metabolism (**a**) PAC production (**b**) PAC-OH production

**Fig. 18:**
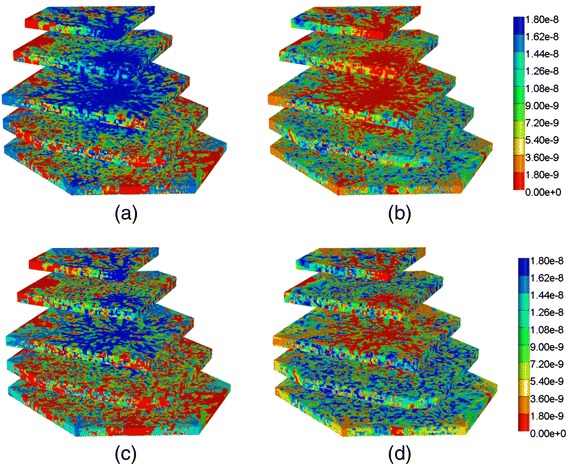
Reactive (6 × 10^−6^ min^−1^) PAC and PAC-OH profiles at 1 min across the lobule without diffusion effects and normal versus low concentration-zonation metabolism in 3D – clipped view. **a** PAC (normal concentration), **b** PAC-OH (normal concentration), **c** PAC (low concentration), **d**PAC-OH (low concentration). Color bar is in mole fraction

To further confirm these comments, we have rerun the models with a lower PAC metabolism rate. Figure 17 compares reactive PAC and PAC-OH production profiles with no diffusion contributions to the flow at these lower reaction rates (6 × 10^−6^ min^−1^). Again only ports A and C are shown. For these cases, the low concentration zonation production is again essentially identical to the no zonation case, but the normal zonation production differs more substantially from the other two cases. This behaviour is reflected in the spatial distributions of PAC and PACOH at various time points as well. Figure 18 compares the final (at 1 min) spatial distributions for PAC and PACOH for the normal and low-oxygen concentration generated CYP distributions. (The no zonation case is essentially identical to the latter case plots, not shown). The normal oxygen generated CYP distribution results in less PACOH conversion.

## Conclusions

### Generalized conclusions relative to our earlier work

The increased morphological variability of the sinusoidal networks generated in this work allows various generalized observations relative to our earlier work on idealized lattice models:

- In 2D due to randomness of the sinusoid patterns, individual producing ports (hepatic veins) respond differently (i.e. at different times) to injected drug. This is similar to the randomness we generated via multiple realizations to our individual well pair models with our previous idealized modelling work. If we sum the individual hepatic vein responses we get an overall 2D lobule drug response (see Fig. 5), the net result of which is an increased spreading (effective diffusion) of the average produced profile.
- Multiple 2D morphological sinusoid realizations can be explored to ascertain the effects of structural variations, as indicated in Table 2. Additionally a 3D model viewed as interacting stacks of 2D sinusoid realizations represents an enhanced method to consider such variability.
- In 3D, because the individual producing ports are completed throughout the vertical extent of the lobule, the variation of response of individual producing ports is seen to be less extreme, although the production curves themselves are more diffuse in 3D than in 2D. Furthermore, the average lobule production summed over all hepatic veins is also more diffuse. Thus the net effect of all these variations is to make the overall response more smooth (i.e. more robust to individual variations).
- When the effect of drug processing (CYP) zonation is included, the effect on drug production characteristics appears quite small and only when the further (smoothing) effects of diffusion are ignored. However, within a lobule we expect there are conditions under which there can be noticeable differences in drug distribution due to zonation. These observations are consistent with our earlier idealized models of zonation. Finally we should emphasize that this treatment of zonation of drug metabolism can be readily extended to other metabolic zonation phenomena occurring in the liver, such as carbohydrate metabolism [21], nitrogen metabolism [22], etc. O_2_ distribution is implicated as a fundamental cause of such zonation in all cases.

### Follow-up work and future directions

Since various liver diseases can be thought to produce structural variations in the lobule, our analysis also gives insight into the role of disease on liver function and performance. In our previous work on variable lattice lobule models [9], we utilized random sinusoid permeability models and percolation concepts to capture some of the structural effects of hepatitis and cirrhosis. We next plan on extending such analysis to our more physiologically realistic 3D lobule model.

It should be mentioned that there exist other computer-generated liver models that attempt to provide a realistic representation of the liver vasculature for blood flow and metabolic reaction simulations. Schwan et al. [23] used an image of a mouse liver produced with a CT scanner. Based on this image data, they reconstructed the structure of the fine branches of the liver vessel system. The liver was then split into 50,000 small blocks. The results of the simulation show that blood flow and metabolic reactions can be tracked in detail on the computer monitor. In a follow-up publication [24] a multi-scale model was generated that links together four scales of modeling. Thus the focus of these authors is the full liver scale (organ model) and not the lobule scale (tissue level) which is the subject of our current paper. In addition, we have also recently developed a spatial model of the full liver (White D, Rezania V, Coombe D, Tuszynski J: Building a 3D virtual liver: a preliminary example – methods for describing vascular generation, blood flow calculations, and hepatic clearance, submitted) utilizing a computational algorithm for generating vasculature and where we have considered upscaling from our lobule model and some comparisons with the work of Schwen et al. [23]. Eventually, our overall modeling approach is intended to provide input data regarding liver metabolism for multi-compartment physiologically-based pharmacokinetic models such as those reviewed by Jones et al. [25]. This could lead to more accurate and individualized predictions for the pharmacokinetic analyses of drugs and drug combinations. It is also of interest to explore the possibility of fractal vascular structures and their consequences on the scaling laws in liver function as reviewed extensively by Pang et al. [26].

Our focus will continue to be on the structural and metabolic changes induced by liver disease and cancer, and its remediation via drug treatments. More particularly, we will focus on drug combination therapy for cancer (e.g. paclitaxel with doxorubicin) [27].

## Figures and Tables

**Fig. 19:**
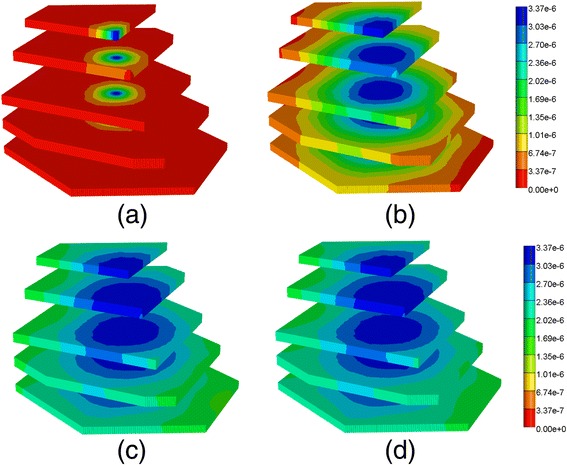
Reactive O_2_ profiles across the lobule with diffusion effects for “normal” injected O_2_ level in 3D – clipped view. **a** O_2_ at 0.01 min, **b** O_2_ at 0.1 min, **c** O_2_ at 0.5 min, **d** O_2_ at 1.0 min. Color bar is in mole fraction

**Fig. 20:**
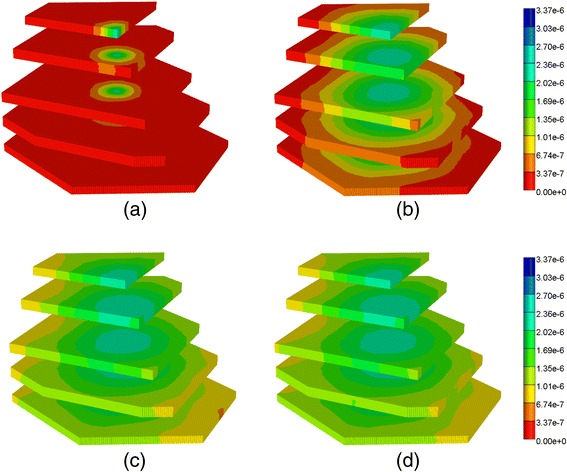
Reactive O_2_ profiles across the lobule with diffusion effects for “low concentration” injected O_2_ level in 3D – clipped view. **a** O_2_ at 0.01 min, **b** O_2_ at 0.1 min, **c** O_2_ at 0.5 min, **d** O_2_ at 1.0 min

**Fig. 21:**
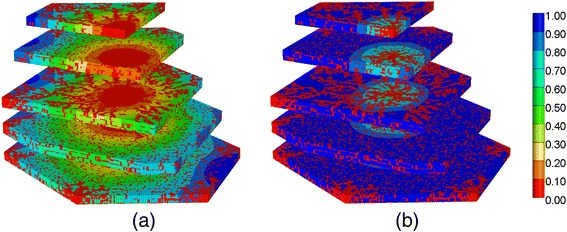
Predicted CYP profiles across the lobule for injected O_2_ level in 3D – clipped view. **a** Normal concentration, **b** low concentration. Color bar is normalized

## Appendix A

### Reactive O_2_ distribution in 3D – indicator of zonation

Here we utilize known O_2_ flow characteristics to predict steady state O_2_ distributions across our mathematically constructed 3D liver lobule. The O_2_ reactive flow characteristics are taken from Davidson et al. [28], and are summarized in Table 3. Davidson et al. [29] utilized this data to analyze O_2_ behaviour in their bioartificial liver. Here we will employ the same data to predict O_2_ distribution across our 3D lobule model.

**Table 3:** O_2_ kinetic elimination Michaelis-Menten parameters plus diffusion (converted from Davidson et al. [28], their Table 1)

Parameter	Characteristic unit	STARS unit
Maximum Rate *v*_max_	352 μM/min	6.34 × 10^−6^ mole fraction/min
Half Saturation Constant *K*_m_	6.24 μM	1.12 × 10^−7^ mole fraction
Linear Rate *v*_max_/*K*_m_	56.4 min^−1^	56.4 min^−1^
Sinusoid Effective Diffusion	3.0 × 10^−9^ m^2^/sec	1.8 × 10^−3^ cm^2^/min
Tissue Effective Diffusion	2.0 × 10^−9^ m^2^/sec	1.2 × 10^−3^ cm^2^/min

The O_2_ metabolic reaction used in our model is assumed to be

$$ {\mathrm{O}}_2 + \mathrm{H}\mathrm{E}\mathrm{P}\to 1.778\ {\mathrm{H}}_2\mathrm{O} + \mathrm{H}\mathrm{E}\mathrm{P} $$

This approach preserves mass and assumes a non-specific metabolic consumption of O_2_ by hepatocytes without detailing explicit cellular metabolic products (since water is a universal component in our model). Reactions occur only in tissue areas (hepatocytes HEP act as “catalysts” for this reaction).

With these parameters, the flow of O_2_ across the lobule is calculated in a manner similar to that of reactive PAC. O_2_ levels are due to the relative contribution of convection, diffusion and hepatocyte O_2_ consumption. Note, however, the flow pattern of O_2_ can be expected to be quite different from that of PAC, both because of its reaction rate but also because of its diffusion coefficient, as this is a very small molecule with much higher diffusion than PAC. Here we establish a steady state O_2_ distribution throughout the 3D lobule by observing the long time O_2_ distribution.

Two cases are considered: (a) “normal” O_2_ injection levels, and (b) “low concentration” O_2_ injection levels to represent some nonspecific illness condition, as listed in Table 3. [(a) 90 mm Hg = 3.37 × 10^-6^ mole fraction; (b) 60 mm Hg = 2.25 × 10^−6^ mole fraction]. Figures 19 and 20 illustrate the evolution of O_2_ profiles across the lobule to steady state for these two cases, respectively. Although there are observable concentration differences between the two cases throughout the lobule, the major differences occur near the injection site.

Rescaled versions of these distributions are used to define corresponding predicted CYP distributions. The actual form of this rescaling is

$$ \left[\mathrm{C}\mathrm{Y}\mathrm{P}\right] = 1.0\ \hbox{-}\ \left(\left[{\mathrm{O}}_2\right]\hbox{-}\ {\left[{\mathrm{O}}_2\right]}_{\min}\right)/\left({\left[{\mathrm{O}}_2\right]}_{\max}\hbox{-} {\left[{\mathrm{O}}_2\right]}_{\min}\right) $$

Here [O2] >0 represents the steady state O_2_ concentration level at any location throughout the lobule for each case, while [O_2_]_min_ and [O_2_]_max_ correspond to the non-zero minimum and maximum injected O_2_ concentration level for the “normal” case (90 mm Hg = 3.37× 10^−6^ molefrac), respectively. Figure 21 shows the resultant CYP profiles for each case. First, there are zero CYP expressed in the sinusoids and near the injection end for the “normal O2 generated” zonation. There are higher CYP levels generated near the production ports, but the individual CPY levels depend on the flow distribution to each port (the ports with highest flow have lower CYP levels). In contrast, for the “normal O2 generated” zonation, there is significantly higher levels of CYP predicted and a much more uniform distribution (except closest to the inlet port).

These zonal CYP distributions are used in the main body of the paper to investigate PAC drug zonation effects.

## Additional files

Additional file 1: Figure S1. Non-reactive PAC profiles across the lobule with diffusion. (a) PAC at 0.01 min, (b) PAC at 0.1 min, (c) PAC at 0.2 min (d) PAC at 0.5 min. Color bar is in molfrac. (PPTX 206 kb) Additional file 2: Figure S5. Non-reactive PAC profiles across the lobule without diffusion in 3D – block view. (a) PAC at 0.01 min, (b) PAC at 0.05 min, (c) PAC at 0.1 min (d) PAC at 1 min. Color bar is in molfrac. (PPTX 613 kb) Additional file 3: Figure S2. Non-reactive PAC profiles across the lobule with diffusion in 3D – block view. (a) PAC at 0.01 min, (b) PAC at 0.05 min, (c) PAC at 0.1 min (d) PAC at 1 min. Color bar is in molfrac. (PPTX 431 kb) Additional file 4: Figure S3. Non-reactive PAC profiles across the lobule with diffusion in 3D – sliced view. (a) PAC at 0.01 min, (b) PAC at 0.05 min, (c) PAC at 0.1 min (d) PAC at 1 min. Color bar is in molfrac. (PPTX 335 kb) Additional file 5: Figure S6. Reactive (6 × 10-3 min-1) PAC and PAC-OH profiles across the lobule without diffusion effects and base case metabolism in 3D – block view. (a) PAC at 0.01 min, (b) PAC at 0.05 min, (c) PAC at 0.1 min, (d) PAC at 1 min, (e) PAC-OH at 0.01 min, (f) PAC-OH at 0.05 min, (g) PAC-OH at 0.10 min, (h) PAC-OH at 1 min. Color bar is in molfrac. (PPTX 1050 kb) Additional file 6: Figure S4. Reactive (6 × 10-3 min-1) PAC and PAC-OH profiles across the lobule with diffusion effects and base case metabolism in 3D – sliced view. (a) PAC at 0.1 min, (b) PAC at 0.5 min, (c) PAC at 1 min, (d) PAC-OH at 0.1 min, (e) PAC-OH at 0.5 min, (f) PAC-OH at 1 min. Color bar is in molfrac. (PPTX 378 kb)
