# Supplementary figures and images for: A physiologically-based flow network model for hepatic drug elimination III: 2D/3D DLA lobule models
Source: Theor Biol Med Model. 2016 Mar 3;13:9. doi: 10.1186/s12976-016-0034-5 (PMC4778290; doi:10.1186/s12976-016-0034-5)

## Slide 1
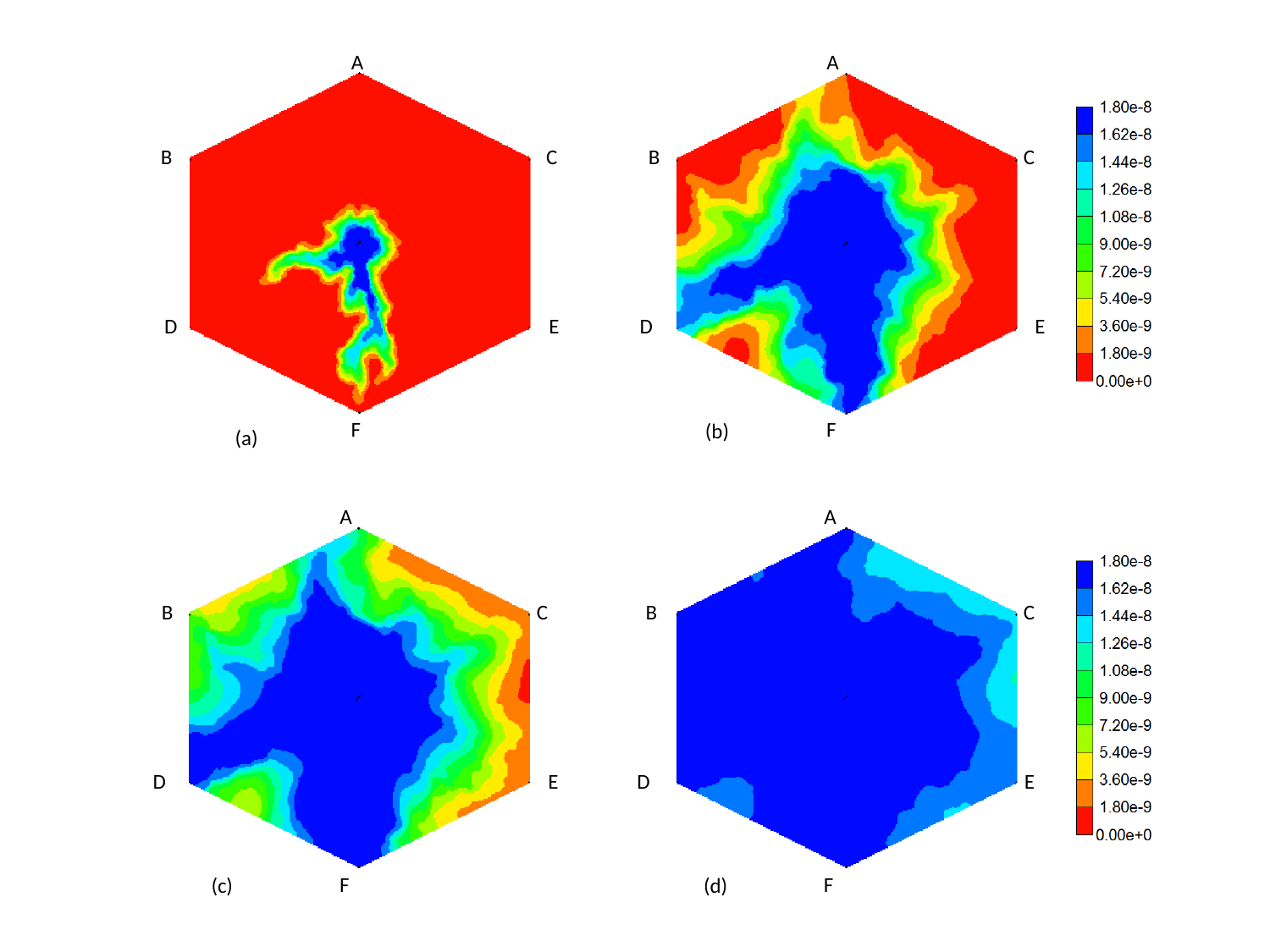

A
C
B
D
E
F
A
C
B
D
E
F
(b)
(a)
A
C
B
D
E
F
A
C
B
D
E
F
(d)
(c)

Supplement: Additional file 1: Figure S1. — Non-reactive PAC profiles across the lobule with diffusion. (a) PAC at 0.01 min, (b) PAC at 0.1 min, (c) PAC at 0.2 min (d) PAC at 0.5 min. Color bar is in molfrac. (PPTX 206 kb) [file 12976_2016_34_MOESM1_ESM.pptx]

## Slide 1
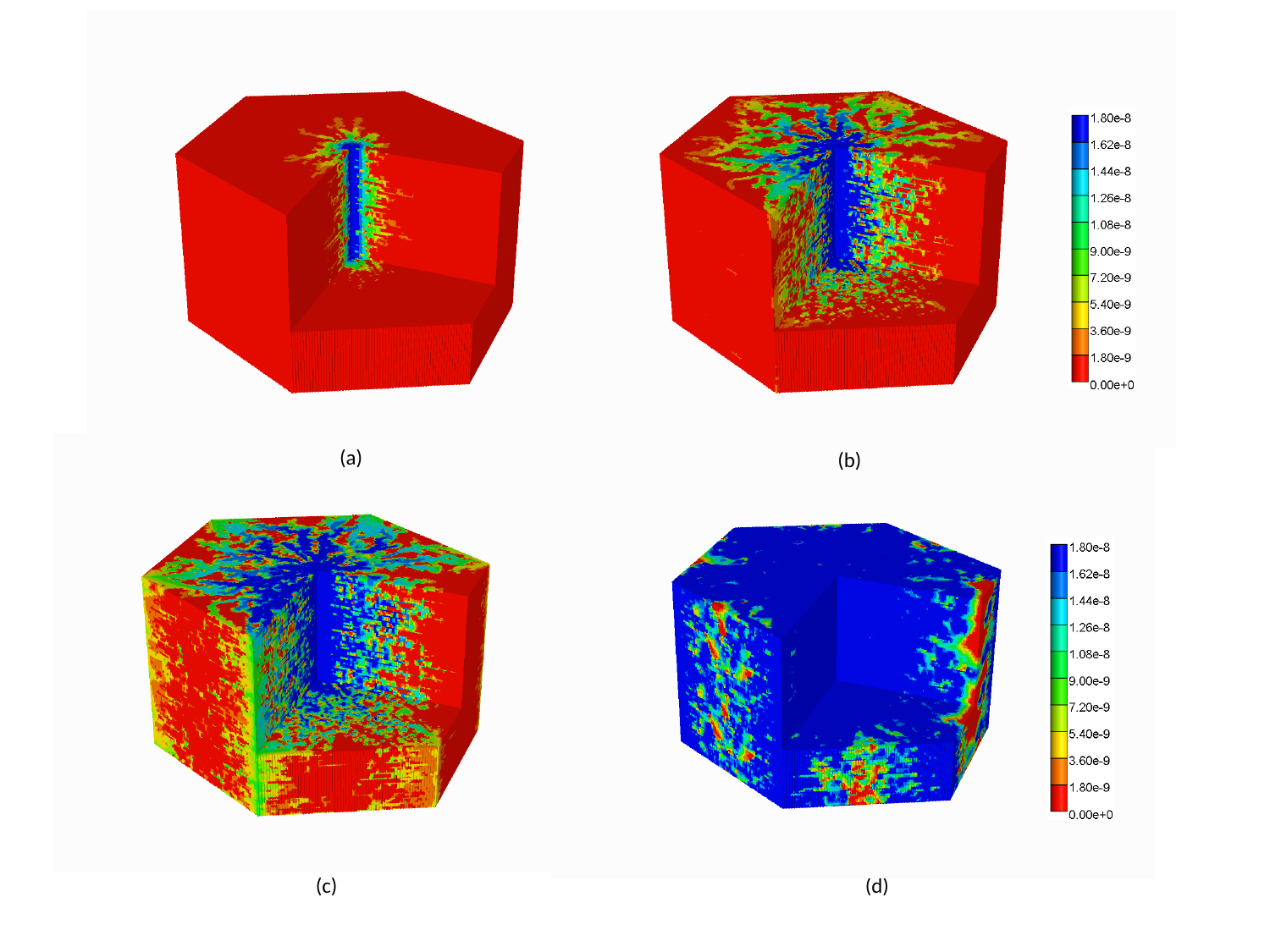

(b)
(a)
(b)
(d)
(c)

Supplement: Additional file 2: Figure S5. — Non-reactive PAC profiles across the lobule without diffusion in 3D – block view. (a) PAC at 0.01 min, (b) PAC at 0.05 min, (c) PAC at 0.1 min (d) PAC at 1 min. Color bar is in molfrac. (PPTX 613 kb) [file 12976_2016_34_MOESM2_ESM.pptx]

## Slide 1
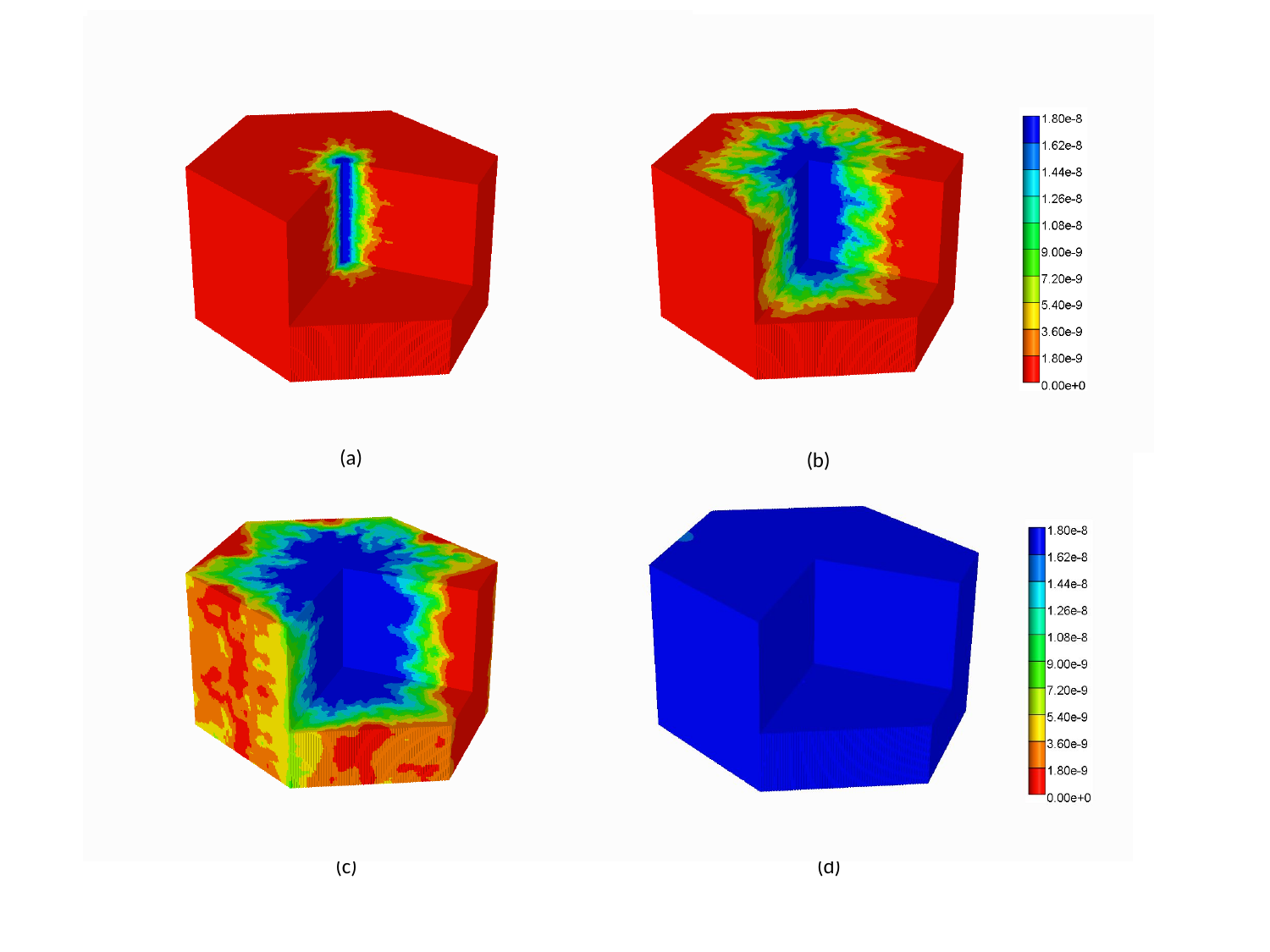

(b)
(a)
(b)
(c)
(d)

Supplement: Additional file 3: Figure S2. — Non-reactive PAC profiles across the lobule with diffusion in 3D – block view. (a) PAC at 0.01 min, (b) PAC at 0.05 min, (c) PAC at 0.1 min (d) PAC at 1 min. Color bar is in molfrac. (PPTX 431 kb) [file 12976_2016_34_MOESM3_ESM.pptx]

## Slide 1
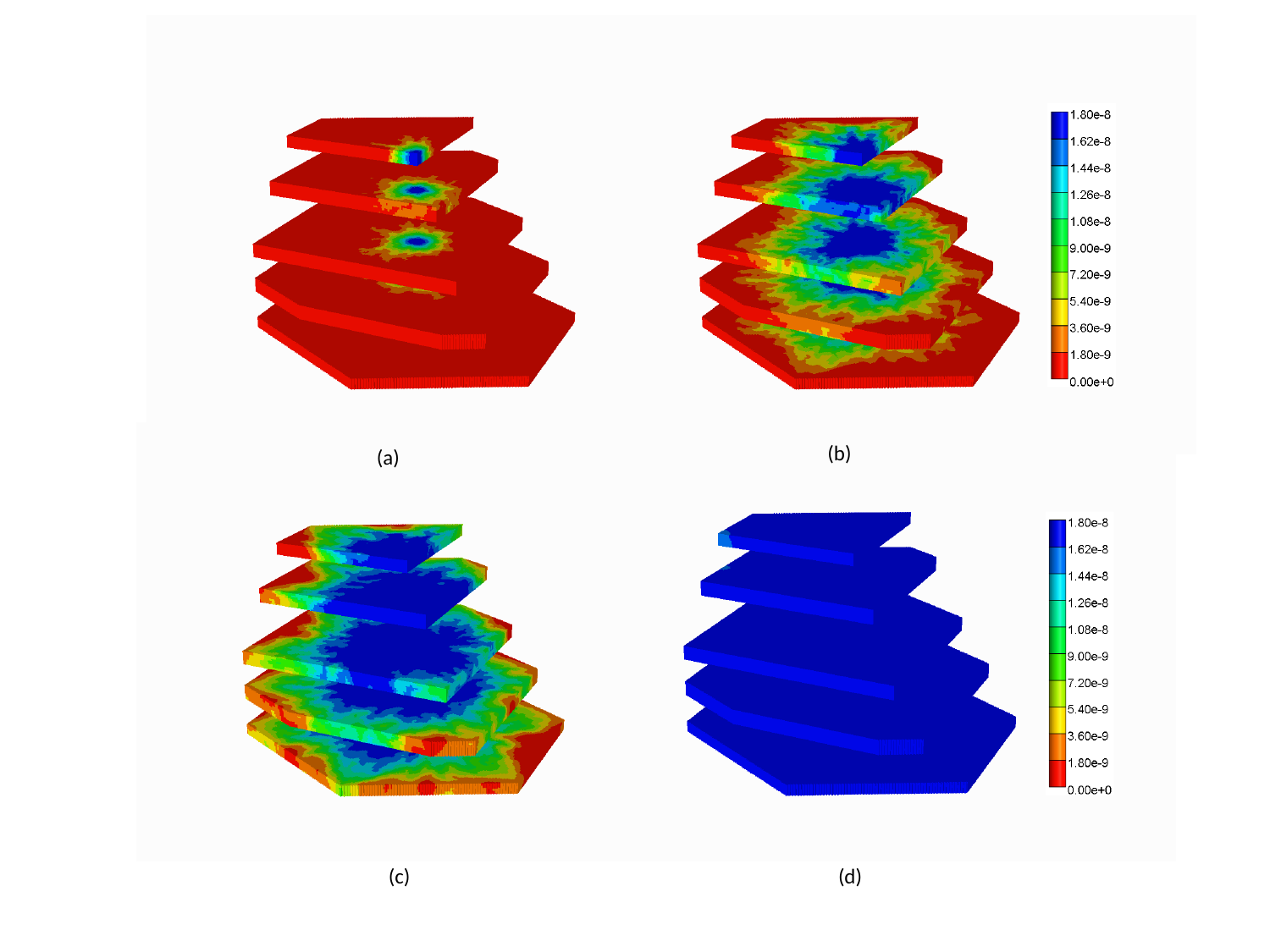

(b)
(a)
(c)
(d)

Supplement: Additional file 4: Figure S3. — Non-reactive PAC profiles across the lobule with diffusion in 3D – sliced view. (a) PAC at 0.01 min, (b) PAC at 0.05 min, (c) PAC at 0.1 min (d) PAC at 1 min. Color bar is in molfrac. (PPTX 335 kb) [file 12976_2016_34_MOESM4_ESM.pptx]

## Slide 1
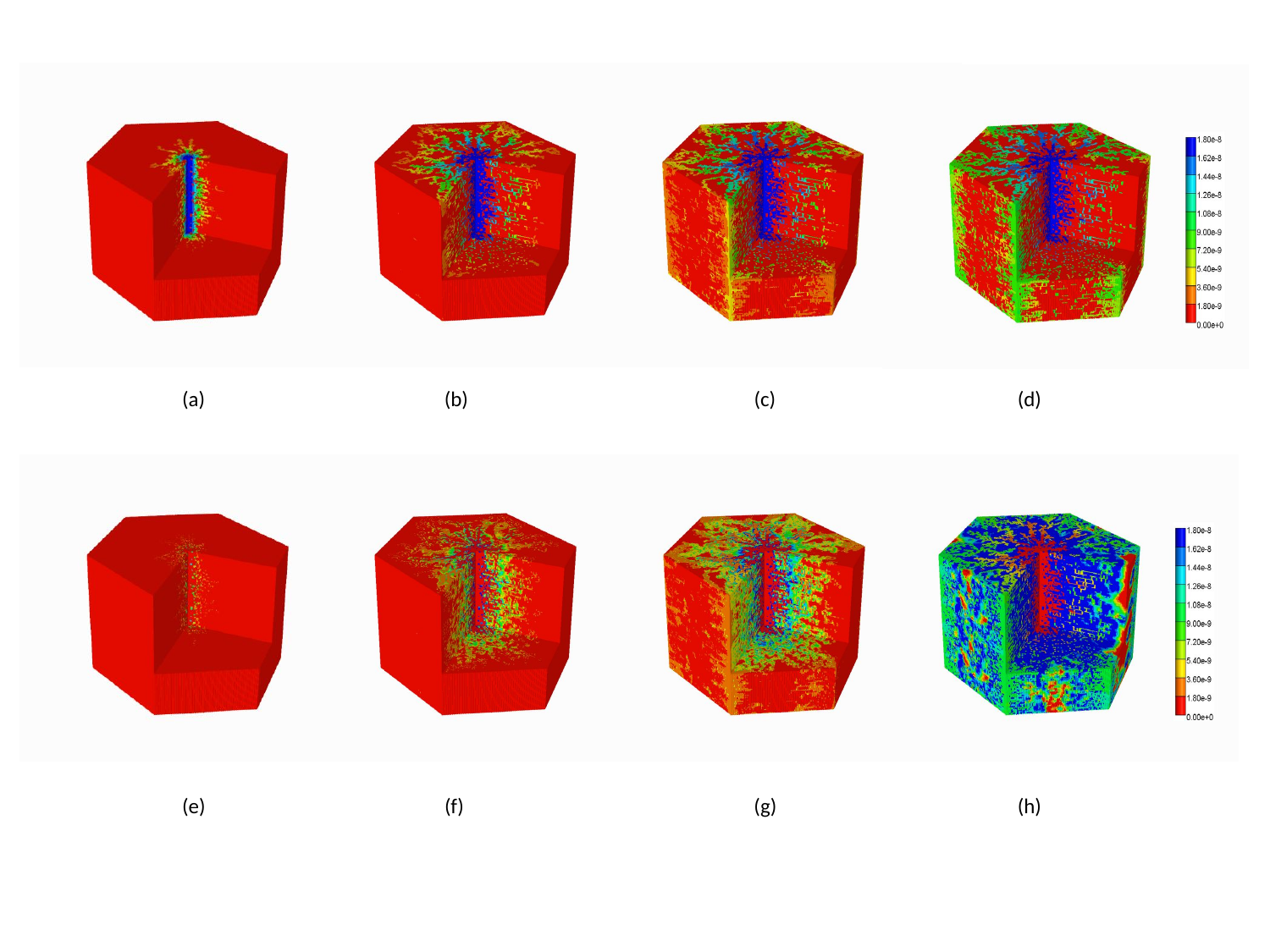

(b)
(a)
(c)
(d)
(f)
(e)
(g)
(h)

Supplement: Additional file 5: Figure S6. — Reactive (6 × 10-3 min-1) PAC and PAC-OH profiles across the lobule without diffusion effects and base case metabolism in 3D – block view. (a) PAC at 0.01 min, (b) PAC at 0.05 min, (c) PAC at 0.1 min, (d) PAC at 1 min, (e) PAC-OH at 0.01 min, (f) PAC-OH at 0.05 min, (g) PAC-OH at 0.10 min, (h) PAC-OH at 1 min. Color bar is in molfrac. (PPTX 1050 kb) [file 12976_2016_34_MOESM5_ESM.pptx]

## Slide 1
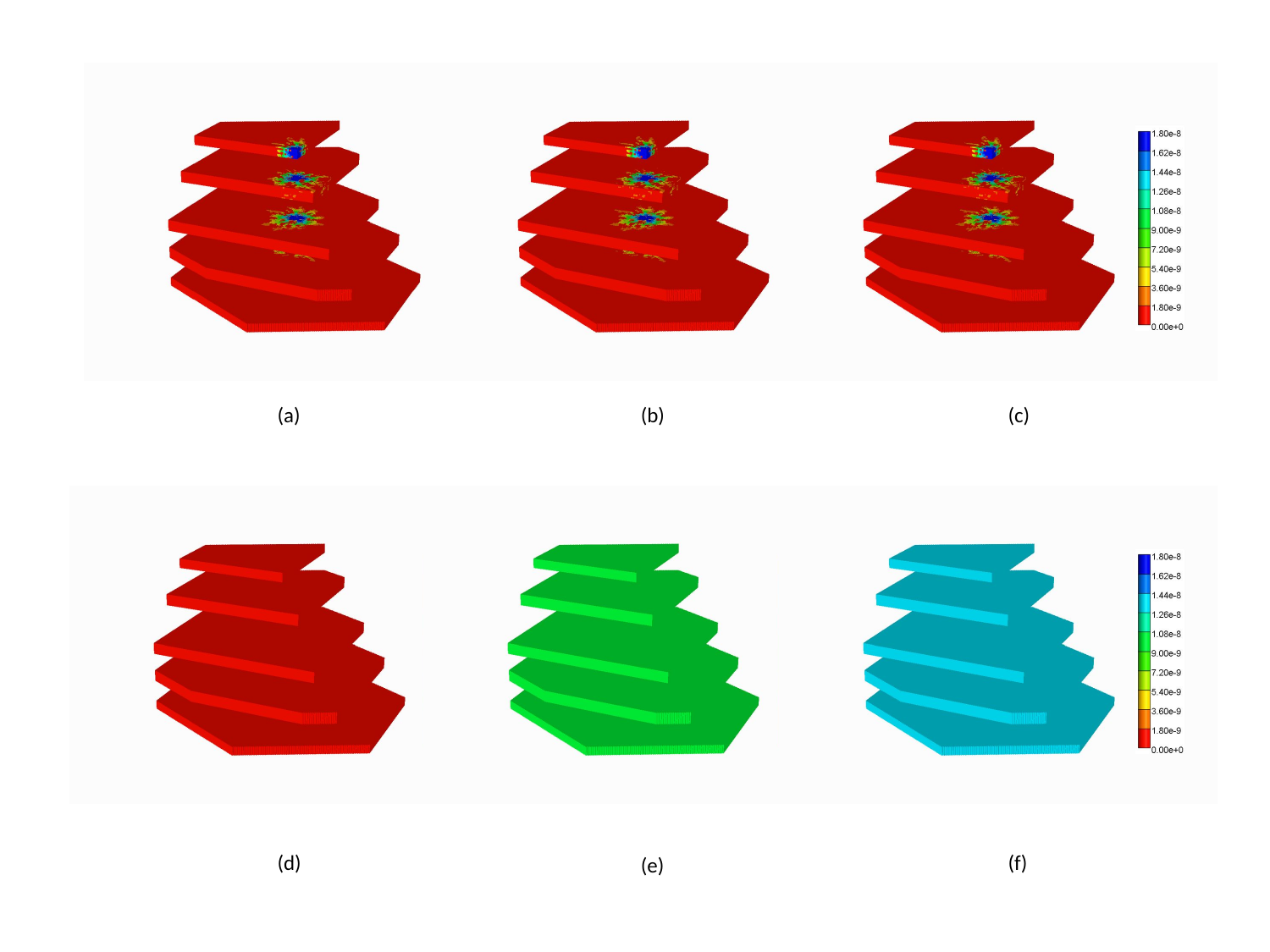

(a)
(b)
(c)
(f)
(d)
(e)

Supplement: Additional file 6: Figure S4. — Reactive (6 × 10-3 min-1) PAC and PAC-OH profiles across the lobule with diffusion effects and base case metabolism in 3D – sliced view. (a) PAC at 0.1 min, (b) PAC at 0.5 min, (c) PAC at 1 min, (d) PAC-OH at 0.1 min, (e) PAC-OH at 0.5 min, (f) PAC-OH at 1 min. Color bar is in molfrac. (PPTX 378 kb) [file 12976_2016_34_MOESM6_ESM.pptx]
